# Metal–Organic Framework Nanomaterials as a Medicine for Catalytic Tumor Therapy: Recent Advances

**DOI:** 10.3390/nano14090797

**Published:** 2024-05-03

**Authors:** Jiaojiao Zhang, Meiyu Li, Maosong Liu, Qian Yu, Dengfeng Ge, Jianming Zhang

**Affiliations:** 1 School of Chemistry and Chemical Engineering, Jiangsu University, Zhenjiang 212013, China; 2 School of Life Science, Jiangsu University, Zhenjiang 212013, China; 2212117002@stmail.ujs.edu.cn; 3 Shengli Oilfield Central Hospital, 31 Ji’nan Rd, Dongying 257034, China; gedengfeng@126.com

**Keywords:** metal–organic frameworks, synergetic therapy, tumor therapy, nanomedicine

## Abstract

Nanomaterials, with unique physical, chemical, and biocompatible properties, have attracted significant attention as an emerging active platform in cancer diagnosis and treatment. Amongst them, metal–organic framework (MOF) nanostructures are particularly promising as a nanomedicine due to their exceptional surface functionalities, adsorption properties, and organo-inorganic hybrid characteristics. Furthermore, when bioactive substances are integrated into the structure of MOFs, these materials can be used as anti-tumor agents with superior performance compared to traditional nanomaterials. In this review, we highlight the most recent advances in MOFs-based materials for tumor therapy, including their application in cancer treatment and the underlying mechanisms.

## 1. Introduction

Cancer, a leading cause of high mortality rates, poses a significant threat to human life [1,2,3]. Timely diagnosis and effective treatment strategies are imperative. The field of nanomedicine has witnessed rapid advancements via the development of sophisticated nanomaterials and a deeper understanding of tumor pathogenesis. The application of nanomaterials in tumor therapy is garnering increased attention [4,5]. Rationally designed nanomaterials can significantly improve the stability of drugs in complex physiological environments and their accumulation at tumor sites by increasing permeability and retention efficacy [6,7,8]. Specifically, the use of metal–organic framework (MOF) nanomaterials has emerged as a promising avenue for drug-free therapeutic diagnostic nanoplatforms in cancer treatment [9].

MOFs are a class of porous crystalline materials with periodic network microstructures constructed by the self-assembly of inorganic metal centers (e.g., metal ions or metal clusters) and organic ligands. The organic ligands and metal centers, acting as the pillars and nodes, respectively, are connected via covalent bonds to form a grid structure with a certain topology. MOFs, as a combination of the rigidity of inorganic materials and the flexibility of organic materials, exhibit exceptional potential across various domains, including optics, catalysis, energy, and biomedicine [10,11], and biotechnology [12,13]. In the realm of biomedicine, MOFs offer numerous advantages, such as high porosity, a large specific surface area, biocompatibility, high drug loading capacity, inherent biodegradability, low cytotoxicity, controlled release patterns, thermochemical stability, and facile surface functionalization [14,15,16]. These characteristics facilitate the integration of functional components like drugs, light/sound sensitizers, nanocatalysts, and targeted molecules for diverse tumor therapies [17,18]. 

**Figure 1:**
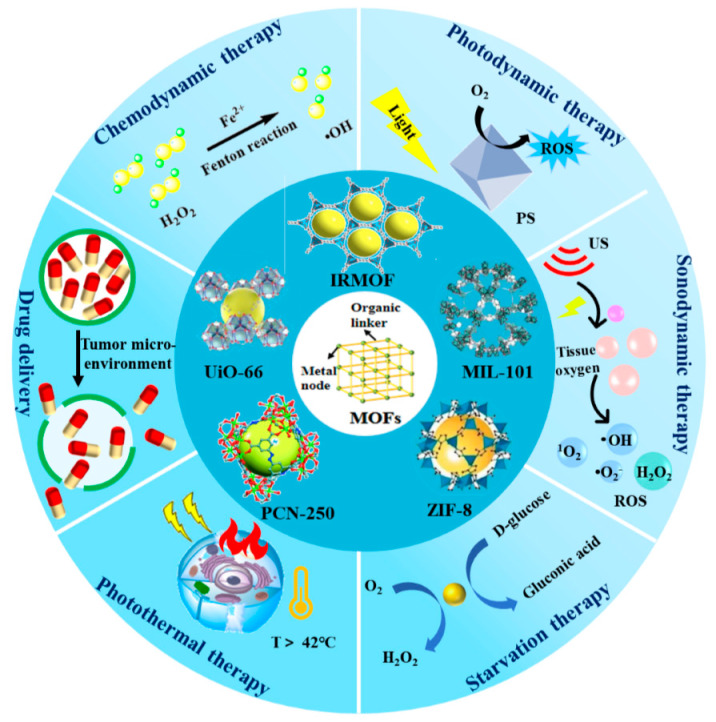
Main scope of the frequently studied MOFs and corresponding various antitumor therapies.

The application of MOFs in cancer therapy represents a rapidly evolving and cutting-edge field within biomedical research. Key challenges and opportunities in MOF-based cancer diagnosis and therapy are being explored, focusing on frequently studied MOFs like Isoreticular MOF (IRMOF), Zeolitic imidazolate framework (ZIF), Materials of Institute Lavoisier (MIL), and porous coordination network (PCN), University of Oslo (UiO) [19]. Additionally, various anti-tumor therapies, such as drug delivery (DD), chemodynamic therapy (CDT), sonodynamic therapy (SDT), photothermal therapy (PTT), photodynamic therapy (PDT), starvation therapy (ST), and multimodal synergistic therapy (MST), have been investigated, elucidating the roles and efficacy of MOFs in these treatment modalities (Figure 1) [20,21,22,23,24,25,26].

This review focuses on recent advances in MOF-based nanomedicine for cancer treatment. It begins with a brief introduction to the unique features of MOFs and subsequently delves into their representative applications in cancer therapy. The review concludes by highlighting challenges in the research domain and proposing intriguing future research directions. The aim is to provide a valuable reference for the development of safe and efficient biomedicines, encouraging broader bio-application of these nanomaterials.

## 2. Properties of MOFs

Various synthetic methods result in diverse morphologies, structures, and pore sizes of metal–organic frameworks (MOFs), influencing their functionalities and potential applications. Commonly employed synthesis techniques encompass the hydrothermal/solvent thermal method, mechanochemical method, ultrasonic synthesis method, microwave synthesis method, ion thermal method, template method, and vapor deposition method [27,28]. By judiciously selecting methods and conditions, targeted performance can be achieved in MOFs. Additionally, these methods can be combined with other manipulations, such as surface modification and drug loading, to further broaden MOFs’ applications in the biomedical field [14].

**Figure 2:**
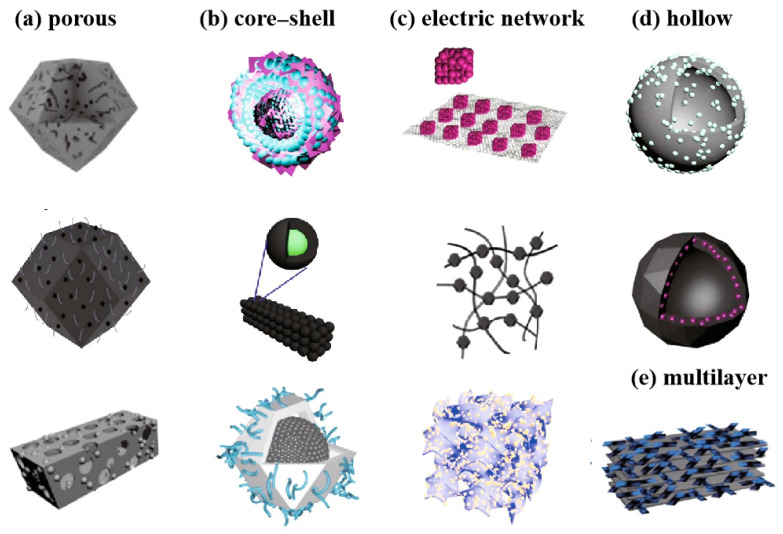
MOFs with different microstructures [33]. Copyright 2022, Springer.

In essence, MOFs are composed of metal ions coordinated with organic ligands, offering controllable size and shape, along with the ability to adjust chemical properties, such as functional groups, acidity, hydrophilicity/hydrophobicity, and surface charge [29]. MOFs exhibit exceptionally high porosity and specific surface area, reaching up to 10,000 m^2^ g^−1^ and a porosity of up to 90% [30]. The extensive surface area and chemical functional groups of MOFs facilitate their modification and functionalization [31]. Some MOFs display piezoelectric responses, converting small pressure changes into significant charge or voltage outputs that interact with mediators in the tumor microenvironment (TME) [32]. Various MOFs and their derived materials with diverse structural morphologies have been designed, including superporous structures, core–shell structures, hollow structures, electric networks, and multilayer structures (Figure 2) [33]. 

The super porous structure comprises multi-stage adjustable pore sizes, such as large pores, medium pores, and micropores, providing MOFs with high specific surface area, pore volume, and good diffusion performance [34,35,36]. Core–shell structures harness the synergistic effect of the core and shell to optimize performance [37,38]. Hollow structures reduce mass transfer distances and offer more active sites for reactions. Moreover, the cavity structure of MOFs can be used to enrich substrates, enhancing the efficiency and selectivity of reactions [39]. The interaction between metal ions and organic ligands in MOFs can regulate electron conduction and charge transport in electrical networks, thereby improving their electrical properties [40,41]. The structural diversity of multilayers provides abundant active sites, larger specific surface area and pore volume, and better chemical stability, compared to the counterparts composed of monolayer and few-layer structures, rendering them a versatile platform for various applications [42].

## 3. Advances in MOFs for Tumor Therapy

### 3.1. MOFs for Drug Delivery

The pore structure of MOFs can be strategically designed to encapsulate and regulate the release rate of drug molecules, enhancing drug stability and mitigating toxic side effects. This renders MOFs a promising DD platform for various cancer therapies [43,44,45]. MOF-based drug delivery can be broadly categorized into three main routes: (1) direct encapsulation of the drug within the pores; (2) covalent coupling of the drug and ligand; (3) drug release from MOFs via a trigger-mediated process [46,47]. 

**Figure 3:**
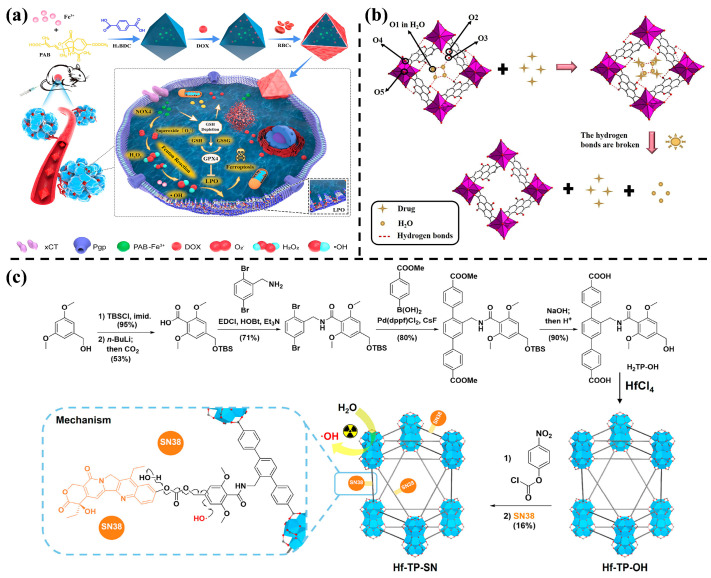
(**a**) Preparation and therapeutic schematic illustration of a multi-drug delivery nanoplatform disguised with MOF membrane [48]. Copyright 2023, Elsevier. (**b**) The light triggered the drug release mechanism of SU-101. (O1-5 represents different oxygen atoms in SU-101) [49]. Copyright 2022, Elsevier. (**c**) Synthesis of Hf-TP-SN-nMOF and release mechanism of SN38 [46]. Copyright 2023, American Chemical Society.

In addressing challenges such as multidrug resistance (MDR), poor cellular uptake, and insufficient intracellular drug release in cancer treatment, innovative approaches have been explored. For instance, Peng et al. [48] developed a defect-rich Fe-based MOF-red blood cell (MOF-RBC) membrane-camouflaged nanoplatform (Figure 3a). Pseudolaric acid B (PAB) acts as an organic connector, encapsulating a ferroptosis drug to trigger ferroptosis and overcome MDR. This MOF-based nanoplatform can chemically degrade in the TME, working in tandem with released Fe^3+^ and PAB to enhance ferroptosis damage. In another study, Zhang et al. [49] synthesized a Bi-based MOF (SU-101) with low toxicity and active photo properties, utilizing ellagic acid (EA) as an organic linker (Figure 3b). Hydrogen bonds between the C=O group in EA and the OH group in ciprofloxacin resulted in an 85.8% loading value of the drug. Moreover, breaking these hydrogen bonds upon light exposure achieved a controlled release rate of up to 95.56%, indicating SU-101’s suitability for drugs that can form hydrogen bonds in drug delivery. Additionally, Xu et al. [46] synthesized a heavy metal-based nanoscale MOF (nMOF; Hf-TP-SN) by covalently bonding organic linkers with 7-ethyl-10-hydroxycamptosine (SN38) prodrugs (Figure 3c). This nanomedicine can be employed in both radiotherapy and chemotherapy (CT), responding to X-ray stimuli.

### 3.2. MOFs for Chemodynamic Therapy

CDT relies predominantly on transition metal ions (Fe^2+^, Mn^2+^, Cu^+^, Co^2+^, etc.), which directly convert overexpressed H_2_O_2_ in tumor cells into highly reactive hydroxyl radicals (•OH) through Fenton or Fenton-like reactions [50,51]. The •OH, as the most active oxygen species (ROS), can effectively kill cancer cells by irreversibly damaging deoxyribonucleic acid (DNA) [52]. Unlike other therapeutic strategies requiring external energy input, CDT is activated by chemical energy conversion within the tumor tissue, avoiding the rapid decay of energy input during treatment [53].

**Figure 4:**
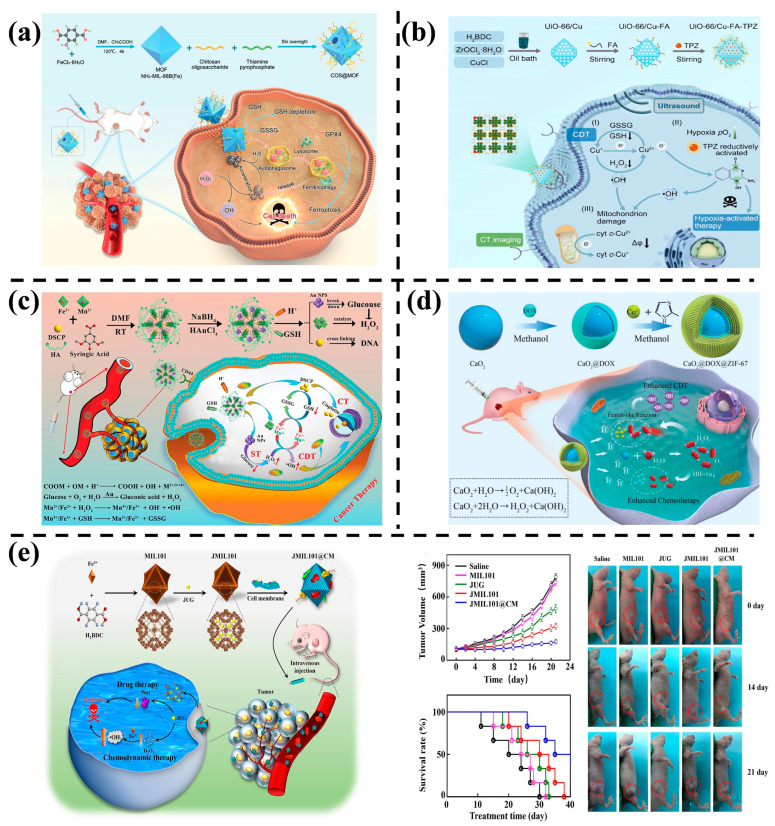
(**a**) The preparation process of COS@MOF and its application in nano-catalyzed tumor therapy [54]. Copyright 2023, Wiley-VCH GmbH. (**b**) Schematic illustration of multiple treatment methods under ultrasound action [55]. Copyright 2023, Elsevier. (**c**) Synthesis method and intracellular mechanism of cascade nanoreactor [56]. Copyright 2023, Wiley-VCH GmbH. (**d**) Synthesis of nanocatalytic drugs and enhancement of CT/CDT [59]. Copyright 2019, Wiley-VCH GmbH. (**e**) Preparation process of nanomedicine and its application in synergistic therapy [52]. Copyright 2022, Elsevier.

MOFs, formed by the self-assembly of metal ions/clusters and organic ligands through coordination bonds, serve as a valuable metal ion source for CDT. Du et al. [54] engineered a synergetic nanocatalytic therapy nanoplatform (COS@MOF) based on a Fe-based MOF, etched by thiamine pyrophosphate (Figure 4a). COS@MOF exhibited an enhanced Fe^2+^/Fe^3+^ ratio and catalytic activity for ROS production, thereby improving CDT efficiency. In addition to Fe ions, Cu ions play a vital role in CDT. In Figure 4b, Cu^+^ was directly doped into the folic acid (FA) modified UiO-66 matrix loaded with a chemotherapy drug (tirapazamine; TPZ). Under ultrasound (US), the rates of Fenton-like reaction and ROS generation increased, enhancing CDT efficiency. Furthermore, US promoted cell death by accelerating drug release and disrupting cell membranes [55]. 

However, the catalytic activity of single metal ions may be insufficient, prompting exploration of the synergistic effect of mixed metal ions for enhanced catalytic ability to generate ROS from H_2_O_2_ in CDT. Chen et al. [56] fabricated Mn/Fe-based MOFs (Figure 4c), releasing Mn^2+/4+^ and Fe^2+/3+^ under the influence of H^+^ and glutathione (GSH) in the TME, thus improving CDT. Metal ions in MOFs, such as Mn^2+/4+^, can react with H_2_O_2_ in a Fenton-like manner, generating cytotoxic •OH that further enhances CDT [57].

To boost CDT efficacy, developing a strategy with highly efficient H_2_O_2_ self-regulating ability is crucial [58]. Gao et al. [59] encapsulated Doxorubicin (DOX) and CaO_2_ nanoparticles (NPs) in a cobalt-based MOF (ZIF-67) (Figure 4d). ZIF-67, being pH-sensitive, decomposes in the acidic tumor environment, releasing Co^2+^ and DOX. CaO_2_ NPs react with H_2_O to form O_2_ and H_2_O_2_, with the generated H_2_O_2_ further catalyzed by Co^2+^ ions to yield highly toxic •OH radicals through a Fenton-like reaction. Simultaneously, the produced O_2_ alleviates hypoxia conditions in tumors, extending the therapeutic efficacy of DOX. In another approach, Zhao et al. [52] introduced biomimetic H_2_O_2_ self-regulating NPs, using a Fe-based MOF (MIL-101) as a carrier for the drug Juglone (JUG) (Figure 4e). In the acidic TME, JUG and Fe^3+^ were cleaved and released. JUG induced apoptosis, increased H_2_O_2_ yield in cells, and Fe^3+^ promoted the conversion of H_2_O_2_ to •OH, accelerating apoptosis and enhancing the anti-tumor effect of CDT.

### 3.3. MOFs for Sonodynamic Therapy

SDT operates in an oxygen-dependent manner, US to induce energy state changes and electron transformations in sonosensitizers, generating ROS that cause irreversible cell damage [60,61,62]. SDT is a novel and attention-worthy approach to cancer therapy, offering deep penetration, in vitro operation, minimal side effects, high precision, and non-invasiveness as an alternative to traditional cancer therapies [63,64,65]. 

**Figure 5:**
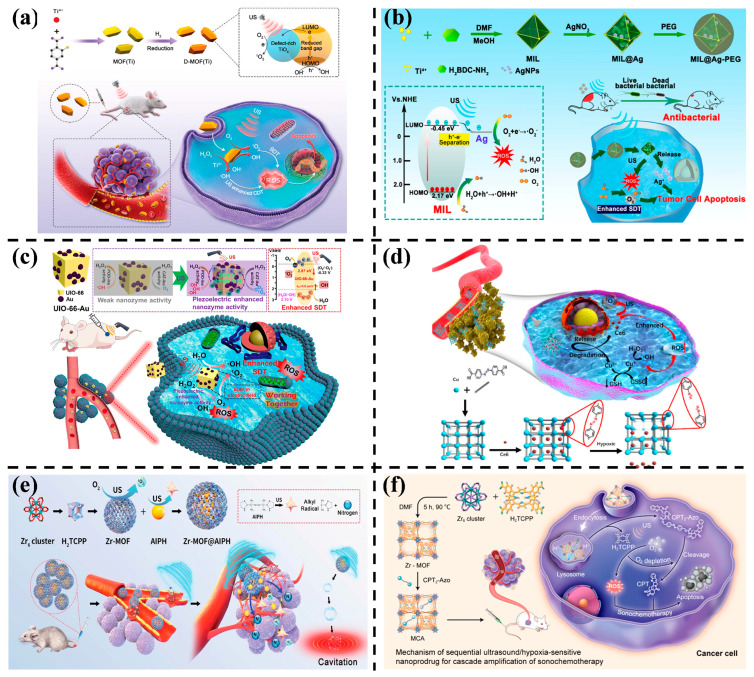
(**a**) Schematic illustration of the preparation process and SDT mechanism of D-MOF(Ti) [66]. Copyright 2021, Wiley-VCH GmbH. (**b**) Schematic illustration of MIL@Ag-PEG synthesis and mechanism of SDT and rapid wound healing [67]. Copyright 2022, American Chemical Society. (**c**) Schematic illustration of UiO-66-Au NPs used to enhance piezoelectric SDT and nanozyme catalytic therapy [69]. Copyright 2023, American Chemical Society. (**d**) Schematic illustration of preparation and cancer therapy of hypoxic responsive Cu-MOF nanosystems [71]. Copyright 2020, Elsevier. (**e**) Schematic illustration of Zr-MOF@AIPH synthesis and anti-tumor therapy [72]. Copyright 2021, Wiley-VCH GmbH. (**f**) The mechanism of SDT and hypoxia-responsive drug therapy under US [73]. Copyright 2022, American Chemical Society.

Liang et al. [66] engineered a defect-rich Ti-based MOF (D-MOF(Ti)) to enhance sound sensitivity for improved SDT (Figure 5a). The narrow band gap (2.49 eV) of D-MOF(Ti) promotes electron-hole separation, boosting ROS yield under US. D-MOF(Ti) also exhibits high Fenton-like activity due to Ti^3+^, achieving CDT. The synergistic effects of enhanced Fenton-like reactions and US acting as an excitation source for SDT contribute significantly to the TME. In another study, Ti-based MOFs (MIL) loaded with Ag NPs (MIL@Ag) demonstrated high electron-hole pair separation efficiency, leading to increased ROS generation under US irradiation (Figure 5b). This enhanced electron transfer and separation efficiency facilitated SDT with a high generation of ROS compared to pure MIL [67].

Piezoelectric MOFs can be employed in SDT, generating an internal electric field under US that favors charge mobility [68]. Deposition of Au NPs on UiO-66 efficiently generated ROS under US, attributed to the internal electric field induced by piezoelectric UiO-66. This enhanced the catalytic efficiency of Au NPs, showcasing the potential for inhibiting solid tumor growth in SDT (Figure 5c) [69]. Hypoxic microenvironments limit the efficacy of SDT. To address this, oxygen-independent free radical generators have been explored [70,71]. Based on this principle, Zhang et al. [71] synthesized a hypoxia-responsive Cu-MOF loaded with sonosensitizers (chlorin e6, Ce6) that can release Cu^2+^ and Ce6 selectively under hypoxic TME. This system remained stable under normal oxygen partial pressure (Figure 5d). The dual effects of Ce6-initiated SDT and Cu^2+^-induced CDT selectively killed cancer cells. Another approach involved constructing a US-responsive dual-sonosensitizer nanoplatform by loading the oxygen-independent free radical generator 2,2’-azobis[2-(2-imidazolin-2-yl)-propane]dihydrochloride (AIPH) on Zr-MOF (Figure 5e). This dual-sonosensitizer nanoplatform improved SDT effectiveness in hypoxic environments and performed well in preclinical and clinical trials for treating pancreatic cancer [72]. The dual-sonosensitizer characteristics of Zr-MOF made it capable of producing singlet oxygen (^1^O_2_) for killing tumor cells when stimulated by US. Moreover, AIPH was decomposed to generate alkyl radicals that can destroy cancer cells even under hypoxic conditions. The decomposition can also generate N_2_ bubbles, which can reduce the cavitation threshold and enhance the acoustic cavitation effect. This, in turn, can facilitate the Zr-MOF@AIPH penetration at the tumor site. 

In a novel strategy, Zhuang et al. [73] synthesized acoustochemical nanotherapeutic prodrug MOF (MCA) NPs. MCA connected a camptothecin (CPT) prodrug with an azobenzene (Azo) bond into the mesopore of an acoustic sensitizer MOF. Under US stimulation, MCA underwent sonosensitizer-mediated SDT, converting O_2_ to cytotoxic ROS. Subsequently, the nontoxic prodrug CPT2-Azo was released, exacerbating the anoxic microenvironment. This process promoted the cleavage of hypoxia-responsive prodrug CPT2-Azo, transforming it from a "non-toxic" to a "toxic" chemotherapy prodrug, ultimately improving therapeutic efficacy (Figure 5f).

### 3.4. MOFs for Photothermal therapy

**Figure 6:**
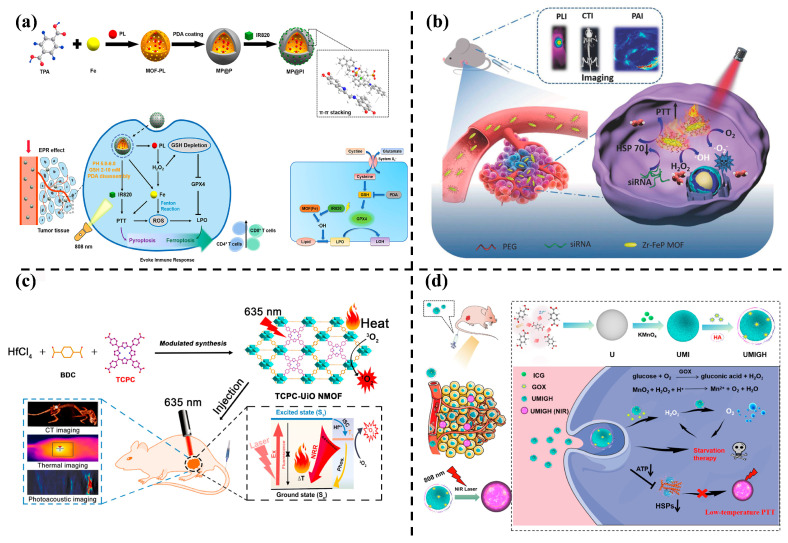
(**a**) Preparation process and application mechanism of MOF nanosystem in vivo tumor therapy [74]. Copyright 2022, American Chemical Society. (**b**) Schematic illustration for multimode imaging diagnosis and combination of HPTT and PDT for cancer treatment [75]. Copyright 2018, Wiley-VCH GmbH. (**c**) Synthesis of TCPC-UiO and its mechanism in antitumor [76]. Copyright 2018, American Chemical Society. (**d**) Synthesis of multifunctional nanoplatform and schematic diagram for enhanced low-temperature PTT and ST [77]. Copyright 2022, Elsevier.

PTT is a tumor hyperthermic ablation strategy that directly kills cancer cells by converting photon energy into heat, offering non-invasiveness and spatiotemporal controllability [74,75,76]. In the context of MOF combination treatment nanosystems, Figure 6a presents polydopamine (PDA)-modified MOF NPs designed for the delivery of the chemical piperlongumine (PL) and photosensitizer (PS) [77]. The MOF serves as an excellent PL carrier and a source of iron donation. The combination of MOF and PL effectively amplifies CDT through the Fenton reaction, resulting in a large amount of ROS. Excess iron, combined with external light and heat, activates the anti-tumor immune response, enhances anti-tumor effects, and induces thermal death of tumor cells. Another multifunctional nanostructure, zirconia-iron porphyrinic metal–organic skeleton (Zr-FeP MOF), generates abundant ROS, including •OH and ^1^O_2_ under near-infrared (NIR) laser irradiation, with a high photothermal conversion efficiency of 33.7%, efficiently inhibiting tumor growth both in vitro and in vivo (Figure 6b) [78]. 

Optically active hydrogen tetrachloride (TCPC) ligands assembled into the prototype structure of Hf-UiO-66, as shown in Figure 6c, resulted in TCPC-UiO with highly efficient photodynamic and photothermal therapeutic effects. The Hf element, with high photothermal conversion efficiency, good photostability, and biocompatibility, as well as strong X-ray attenuation ability, endowed TCPC-UiO with significant anti-tumor effects in in vivo experiments on H22 tumor-bearing mice, achieving a tumor inhibition rate of up to 90% [79].

Hypothermic PTT (HPTT), a non-invasive treatment method, is depicted in Figure 6d. Zhang et al. [80] decorated UiO-66 with the photothermal agent indocyanine green (ICG) through physical adsorption, encapsulated it with a biodegradable MnO_2_ layer (UMI), and attached catalytically active enzyme glucose oxidase (GOx) to the UMI NPs. Upon 808 nm laser irradiation in in vitro tests, the intracellular adenosine triphosphate (ATP) decreased due to the photothermal effect of ICG. The introduction of GOx further reduced ATP levels, diminishing heat shock proteins (HSPs)-mediated heat resistance. The MnO_2_ layer decomposed H_2_O_2_ into O_2_ in the tumor, enhancing the effects of HPTT and starvation therapy (ST).

### 3.5. MOFs for Photodynamic Therapy 

PDT involves using irradiated light of a specific wavelength to activate PS, which releases cytotoxic ROS via photochemical reactions, inducing apoptosis or necrosis in tumor tissue cells and triggering cell death [78]. Porphyrin, acting as a metal–organic backbone for organic linkers, serves as a PS and PDT carrier. Porphyrin-based PS-modified multifunctional MOF NPs can effectively avoid PS accumulation, significantly improving PDT accuracy [79]. Designed with tunable structures and high porosity, porphyrin-based MOFs integrate PSs in periodic arrays, making them promising therapeutic nanoagents for cancer PDT [80]. 

**Figure 7:**
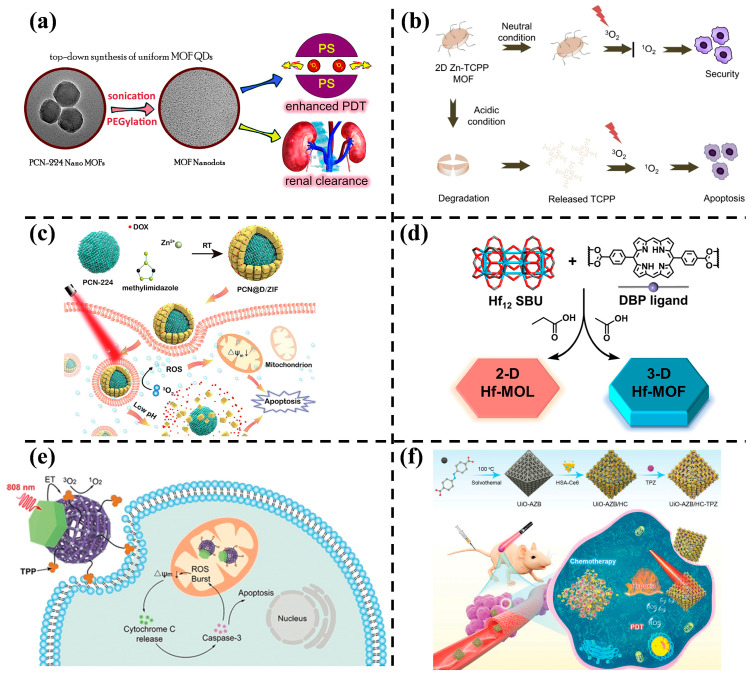
(**a**) Design of MOF quantum dots and schematic illustration of their use to enhance PDT [81]. Copyright 2019, American Chemical Society. (**b**) The mechanism of 2D Zn-TCPP MOF in vivo PDT [82]. Copyright 2021, Springer. (**c**) Schematic illustration of CN@D/ZIF NPs synthesis and PDT/CT combined therapy [83]. Copyright 2023, Elsevier. (**d**) The structures of Hf-MOL and Hf-MOF [84]. Copyright 2022, American Chemical Society. (**e**) Mechanism illustration of upconversion MOFs activated by 808 nm NIR and mitochondria-targeted enhancement of PDT [85]. Copyright 2020, Wiley-VCH GmbH. (**f**) Synthesis of nanotherapeutic system and photo-activated hypoxia-responsive of tumor DD schematic [86]. Copyright 2022, Wiley-VCH GmbH.

Wang et al. [81] prepared ultrasmall porphyrin-based MOF quantum dots (MOF QDs) using a liquid phase stripping method (Figure 7a). MOF QDs generated twice as much effective ROS as MOF NPs under the same light irradiation conditions. Controlled experiments demonstrated significantly prolonged survival time in mice treated with a combination of MOF QDs and light, showcasing the favorable PDT efficacy of MOF QDs in cancer therapy. Additionally, the crystal structure of MOF QDs influenced renal clearance and tumor accumulation capacity in vivo. Tetrakis(4-carboxyphenyl)porphyrin (TCPP) is an efficient PS producing ^1^O_2_. Hang et al. [82] prepared a two-dimensional (2D) porphyrin-based MOF (Zn-TCPP MOF) as PS, achieving controlled photodynamic properties from neutral to acidic environments (Figure 7b). In an acidic environment, the decomposed MOF structure released TCPP, effectively generating ^1^O_2_, inducing cancer cell apoptosis, and enhancing photodynamic activity.

Liu et al. [83] reported a core–shell nanohybrid by assembling DOX-encapsulated ZIF-8 on the surface of Zr (IV)-based porphyrinic MOFs (labeled as PCN@D/ZIF) (Figure 7c). The acidic pH-response degradation of ZIF-8 promoted DOX release (>80%) in the acidic TME. Porphyrinic MOFs, when exposed to light, produced abundant ^1^O_2_, enhancing CDT/PDT therapeutic efficacy. Luo et al. [84] revealed a significant enhancement of PDT efficacy by downscaling the size of PS from three-dimensional (3D) MOF NPs to 2D nano metallic organic layers (MOLs) (Figure 7d). MOLs with a monolayer structure produced more ROS and exhibited significantly higher cytotoxicity than MOF NPs. In a mouse model of triple-negative breast cancer, Hf-MOLs showed a 49.1% greater tumor inhibition, an 80% higher cure rate with 16.3-fold lower metastasis potential than Hf-MOF NPs.

As shown in Figure 7e, a NIR light-activated PDT nanoplatform was designed based on porphyrinic MOFs and upconversion (UC) NPs, combined with a mitochondrial targeting strategy. This nanostructure integrated the advantages of efficient energy transfer from UCNPs to MOF domains and surface functionalization with triphenylphosphine (TPP), a mitochondrial targeting ligand, for a targeted effect. The nanoplatform allowed the generation of ^1^O_2_ with an ablation of an 808 nm laser to minimize the overheating effect. Importantly, mitochondria-targeted PDT initiated the intrinsic apoptotic pathway, resulting in superior therapeutic efficacy over non-targeted treatments [85]. 

A hypoxia-responsive MOF containing an azobenzene group as an organic linker (UiO-AZB) was reported [86]. In this system, the surface of UiO-AZB was modified with chlorin e6 (Ce6)-conjugated human serum albumin (HSA), and tirapazamine (TPZ) was encapsulated into UiO-AZB as a hypoxia-activated prodrug. The resulting nanosystem (UiO-AZB/HC-TPZ) efficiently produced ^1^O_2_ upon 660 nm light irradiation and induced severe hypoxia in tumors. This process (Figure 7f) triggered the degradation of the frameworks and the controlled release of activated TPZ for chemotherapy, leading to improved antitumor treatment through the combination of PDT and hypoxia-activated chemotherapy.

### 3.6. MOFs for Starvation Therapy

ST is a is a novel approach utilizing MOFs-based nanomedicines to treat cancer by introducing glucose oxidase (GOx) into the tumor site. This process aims to consume glucose within the tumor, depriving tumor cells of their energy supply and leading to cell death due to starvation [87,88]. In the ST system, GOx catalyzes the conversion of glucose to highly reactive gluconic acid and H_2_O_2_, and these chemicals continue to activate CT and CDT modes [89,90].

**Figure 8:**
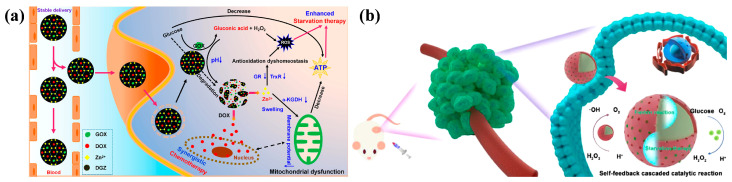
(**a**) Schematic illustration of enhanced ST synergistic CT with mitochondrial dysfunction and antioxidant imbalance [91]. Copyright 2022, American Chemical Society. (**b**) Schematic illustration of FMG enhancing CDT and accelerating ST synergistic cancer therapy [92]. Copyright 2023, Elsevier.

For example, Huo et al. [91] integrated Zeolitic Imidazolate Frameworks (ZIFs) into GOx and DOX, effectively protecting GOx activity for DD (Figure 8a). GOx enzymes played vital roles in inhibiting mitochondrial energy metabolism and promoting ROS accumulation by facilitating glucose acidification, degradation of MOF, and subsequent release of Zn^2+^, consequently enhancing the synergistic effect of ST combined with CT. Additionally, Ni et al. [92] proposed a nano pH-responsive intelligent Fenton configuration with the synergistic effect of ST by encapsulating GOx onto a Fe_3_O_4_@MIL-100 heterojunction (denoted as FMG). The encapsulated GOx successfully activated the ST process and further enhanced the CDT efficiency (Figure 8b).

### 3.7. MOFs for Multimodal Synergistic Therapy 

Currently, addressing the complex cancer system is challenging for a single therapeutic modality due to the complexity, heterogeneity, and diversity of TMEs. Consequently, cancer treatment has evolved from a past single treatment model to the current paradigm of MST to improve therapeutic efficacy. The integration of multiple therapeutic modalities onto a single nanoplatform is promising and represents emerging nanotechnology, as opposed to simply physically mixing modalities to achieve a simple additive therapeutic effect [93,94,95].

**Figure 9:**
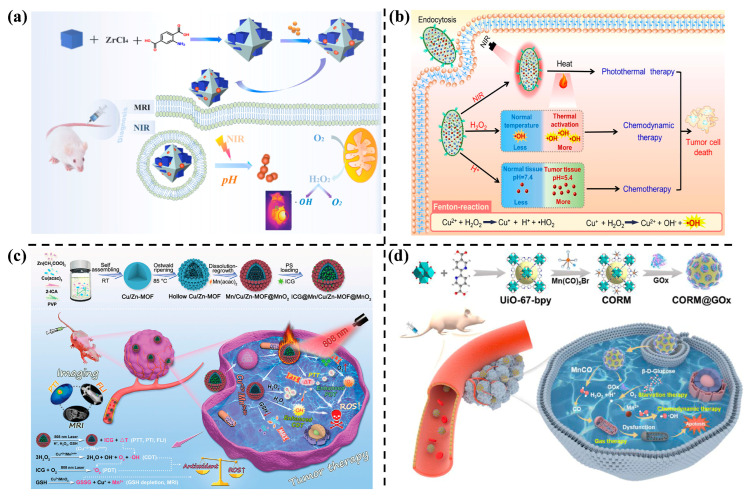
(**a**) Schematic illustration of synthesis and synergistic therapeutic strategy of UiO-66-NH_2_/PB [96], Copyright 2021, Elsevier. (**b**) Schematic illustration of the synthesis of DOX/CuS@Cu-MOF/PEG and its application in PTT/CDT/CT synergistic therapy [97]. Copyright 2022, Elsevier. (**c**) Schematic illustration of PTI/FLI/MRI combined with PTT/PDT/CDT treatment [98]. Copyright 2021, Wiley-VCH GmbH. (**d**) Synthesis of H_2_O_2_-responsive nanoplatform and its application in ST/CDT/GT synergistic therapy [57]. Copyright 2022, Elsevier.

For instance, Gao et al. [96] modified Prussian blue (PB) on the surface of UiO-66-NH_2_ (UiO-66-NH_2_/PB) for imaging-guided synergistic therapy involving CT, PTT, and CDT (refer to Figure 9a). This hybrid serves a dual functionality as a drug carrier, loading DOX via hydrogen bonding and releasing it in the acidic medium of tumors due to protonation. Additionally, it functions as a Fenton-like agent, eliminating cancer cells by catalyzing endogenous H_2_O_2_ into •OH in tumor cells. This process is accompanied by the generation of O_2_, which regulates hypoxia and enhances the anticancer efficiency of released DOX.

In another endeavor, Geng et al. [97] prepared a CuS@Cu-MOF/PEG nanocomposite serving as an integrated thermosensitive nanoplatform (Figure 9b). This nanohybrid exhibits distinct characteristics compared to previous MOF nanoagents: (1) a wider NIR absorption wavelength and higher thermal conversion efficiency for PTT; (2) characterized by Fenton-like reactions, it exhibits higher photothermal effects and accelerates the rate of therapeutic response; (3) the large surface area allows for high DOX-loading ability, facilitating CT; (4) tumors can be monitored by photoacoustic and thermal imaging after injection of the nanohybrids. In sum, the synergistic effects of PTT/CDT/CT inhibit tumor growth, making the CuS@Cu-MOF/PEG nanocomposite an effective multifunctional theranostic nanoagent for MST.

Similarly, as shown in Figure 9c, the integration of mixed Mn, Cu, Zn, and MnO_2_ in the MOF (Mn/Cu/Zn-MOF@MnO_2_) has been employed to load ICG as a photosensitizer for collaborative PTT, PDT, CDT, and guided multimodal imaging [98]. In this system, the mixed-valence metal ions undergo a Fenton-like reaction for CDT, catalyzing the generation of O_2_, relieving tumor hypoxia, and depleting GSH. This results in a ‘turn on’ magnetic resonance imaging (MRI). ICG enclosed in the hollow structure enables photothermal conversion for photothermal imaging and PTT. Once released at the tumor site, fluorescence imaging and enhanced PDT with self-supplied O_2_ are observed from ICG.

Gas therapy (GT), as a rapidly emerging therapeutic modality, has been widely explored in the biomedical field. In this context, Wang et al. designed a unique H_2_O_2_-responsive CO gas generator for synergistic therapy (Figure 9d). Manganese carbonyl (MnCO) and GOx were loaded on Zr-based MOFs (designated as UiO-67-bpy). GOx can consume glucose to produce gluconic acid and H_2_O_2_, leading to ST. Meanwhile, H_2_O_2_ can cause the release of CO, which damages mitochondria and leads to apoptosis of tumor cells. The Mn^2+^ species involved in CDT produce highly toxic •OH through Fenton-like reactions. In vitro and in vivo studies have shown that the multifunctional CO gas-controllable release nanoplatform (CORM@GOx) achieved the combined ST/CDT/GT effect [57].

## 4. Summary and Outlook

This paper outlines recent advances in MOFs as nanomedicine for tumor treatment, via DD, CDT, PTT, PDT, SDT, ST, and MST. The preponderance of MOFs is featured with their unique structural characteristics, multifunctionality, efficient loading capacity, and precise targeting. These advantages enable broad application prospects for MOFs as nanomedicine. However, to realize the full potential of MOFs in tumor therapy, it is essential to address some key challenges, such as the safety, immune response, targeting accuracy, morphology, and large-scale synthesis of MOFs.

First, like other nanomaterial systems for bio-research, their long-term in vivo safety and toxicity need to be fully explored. These materials usually undergo a slow degradation process and cannot be easily excreted from the body, presenting a potential bio-risk. Currently, the therapeutic effect of MOFs has only been studied at the cellular and animal levels. Most studies emphasize the short-term toxicity, ignoring the long-term and acute toxicity of MOFs, which is not conducive to the widespread application of MOFs in tumor therapy. Moreover, their accumulation in the organs may result in side effects, which may cause damage to normal cells. 

Secondly, evaluating the biocompatibility and immunogenicity of MOFs is essential. Some MOFs may provoke an immune response by themselves or through their degradation products in the body. Therefore, understanding the complex interactions between MOFs and the immune system is critical for optimizing their use in biomedical applications.

Thirdly, the challenge of targeting accuracy should not be overlooked. The stability and drug release behavior of MOFs can be affected by dynamic changes in the internal environment, such as pH, temperature, and ion concentration, during the delivery of therapeutic drugs to target tissues. This necessitates extensive research into surface modification, ligand selection, and drug release mechanisms to enhance the targeting precision of MOFs, ensuring that drugs are delivered accurately and effectively to the tumor site while minimizing effects on non-target tissues.

Fourth, the influence of the MOF morphology or structure on the material–cell interaction should be optimized to enhance the overall therapeutic results. In general, the materials with a size <100 nm are favorable for endocytic cell uptake and the following drug transport or dynamic treatment process intracellularly. Larger MOFs might be retained within the body for extended periods, potentially leading to accumulation in organs and compromising organ function. It is advisable to select or optimize the size of MOFs based on the characteristics of tumor cells to increase therapeutic efficacy. Ultra-small or 2D nanostructures, such as quantum dots or nanosheets, may facilitate stronger material–cell interactions than conventional nanoparticles, potentially leading to enhanced treatment effects. Therefore, innovating the structure or components of MOFs to enhance safety, loading capacity, or activity remains a key research priority.

Lastly, significant efforts should be directed toward developing more economically viable techniques for synthesizing MOFs on a large scale. The synthesis process, affected by factors like solvents, temperature, and pressure, involves numerous variables including organic ligands, solvent types, and ratios, which influence the controllable design and synthesis of MOFs. While the solvent thermal method is commonly used, there is a need for new, optimized synthesis methods that allow precise control over these factors and variables. This will enhance the reliability and cost-effectiveness of MOF production. To further investigate the properties and performance of MOFs as biomedical agents, developing reliable and cost-effective synthetic strategies is crucial.

In conclusion, despite the challenges, there is optimism that ongoing research will overcome these obstacles. The unique properties of MOFs hold promise for the development of efficient, safe, and economically viable nanomedicine for cancer treatment. Future investigations should contribute to advancing the understanding and applications of MOFs in the field of tumor therapy.

## Data Availability

Not applicable.
